# Burnout and resilience in intensive care Nursing professionals in the face of COVID-19: A multicenter study

**DOI:** 10.1590/1518-8345.5778.3537

**Published:** 2022-05-30

**Authors:** Lizandra Santos Vieira, Wagner de Lara Machado, Daiane Dal Pai, Tânia Solange Bosi de Souza Magnago, Karina de Oliveira Azzolin, Juliana Petri Tavares

**Affiliations:** 1 Universidade Federal do Rio Grande do Sul, Porto Alegre, RS, Brasil.; 2 Bolsista da Coordenação de Aperfeiçoamento de Pessoal de Nível Superior (CAPES), Brasil.; 3 Pontifícia Universidade Católica do Rio Grande do Sul, Faculdade de Psicologia, Porto Alegre, RS, Brasil.; 4 Universidade Federal do Rio Grande do Sul, Departamento de Enfermagem Médico-Cirúrgica, Porto Alegre, RS, Brasil.; 5 Universidade Federal de Santa Maria, Departamento de Enfermagem, Santa Maria, RS, Brasil.

**Keywords:** Burnout, Professional, Resilience, Psychological, Nursing, Intensive Care Units, Coronavirus, Brazil, Esgotamento Profissional, Resiliência Psicológica, Enfermagem, Unidades de Terapia Intensiva, Coronavírus, Brasil, Agotamiento Profesional, Resiliencia Psicológica, Enfermería, Unidades de Cuidados Intensivos, Coronavirus, Brasil

## Abstract

**Objective:**

to analyze the relationship between the Burnout dimensions and the work resilience of intensive care Nursing professionals in the COVID-19 pandemic in four hospitals from southern Brazil.

**Method:**

this is a multicenter and cross-sectional study, composed of 153 nurses and nursing technicians of the Intensive Care Units. Sociodemographic, health and work-related questions were collected, and the Maslach Burnout Inventory and Resilience at Work Scale 20 instruments were applied. The data were submitted to descriptive analysis and to bivariate and partial correlations (network analysis).

**Results:**

work resilience presented an inverse correlation to emotional exhaustion (r=-0.545; p=0.01) and depersonalization (r=-0.419; p=0.01) and a direct one to professional achievement (r=0.680; p=0.01). The variable with the greatest influence on the correlation network was the perception of the impact of the pandemic on mental health.

**Conclusion:**

resilience interferes in the emotional exhaustion and low professional achievement domains of Burnout. Emotional exhaustion is conducted through minor psychological disorders, with an impact on the workers’ physical and mental health variables. The development of institutional resilience should be encouraged in order to moderate the illness.

## Resumo

**Objetivo:**

analisar a relação entre as dimensões do *Burnout* e a resiliência no trabalho dos profissionais de enfermagem de terapia intensiva na pandemia de COVID-19, em quatro hospitais do Sul do Brasil.

**Método:**

trata-se de um estudo multicêntrico, de delineamento transversal, composto por 153 enfermeiros e técnicos de enfermagem das Unidades de Terapia Intensiva. Foram coletadas questões sociodemográficas, de saúde e laborais e aplicados os instrumentos *Maslach Burnout Inventory* e *Resilience at Work Scale* 20. Os dados foram submetidos à análise descritiva e a correlações bivariadas e parciais (análise de rede).

**Resultados:**

a resiliência no trabalho apresentou correlação inversa ao desgaste emocional (r= -0,545; p=0,01) e à despersonalização (r= -0,419; p=0,01) e direta à realização profissional (r= 0,680; p=0,01). A variável com maior influência sobre a rede de correlações foi a percepção do impacto da pandemia sobre a saúde mental.

**Conclusão:**

a resiliência interfere nos domínios desgaste emocional e baixa realização profissional do *Burnout*. O desgaste emocional é conduzido por meio dos distúrbios psíquicos menores, com impacto sobre as variáveis de saúde física e mental dos trabalhadores. Deve-se fomentar o desenvolvimento da resiliência no âmbito institucional, a fim de moderar o adoecimento.

## Resumen

**Objetivo:**

analizar la relación entre las dimensiones del *Burnout* y la resiliencia en el trabajo de los profesionales de enfermería de cuidados intensivos durante la pandemia de COVID-19, en cuatro hospitales del sur de Brasil.

**Método:**

se trata de un estudio multicéntrico, transversal, compuesto por 153 enfermeros y técnicos en enfermería de las Unidades de Cuidados Intensivos. Se recolectaron datos sociodemográficos, de salud y laborales y se aplicaron los instrumentos *Maslach Burnout Inventory* y *Resilience at Work Scale* 20. Los datos fueron sometidos a análisis descriptivo y a correlaciones bivariadas y parciales (análisis de redes).

**Resultados:**

la resiliencia en el trabajo tiene una correlación inversa con el agotamiento emocional (r= -0,545; p=0,01) y la despersonalización (r= -0,419; p=0,01) y directa con la realización profesional (r= 0,680; p=0,01). La variable con mayor influencia en la red de correlaciones fue la percepción del impacto de la pandemia en la salud mental.

**Conclusión:**

la resiliencia afecta los dominios agotamiento emocional y baja realización profesional del *Burnout*. El agotamiento emocional se manifiesta a través de trastornos psíquicos menores e impacta en las variables de salud física y mental de los trabajadores. Se debe fomentar el desarrollo de la resiliencia a nivel institucional para moderar la enfermedad.

**Highlights**

(1) A multicenter study conducted in four reference hospitals in COVID-19 care.

(2) Data from the first contagion peak, with high exposure of the professionals.

(3) An unprecedented study, with advancement of knowledge about work resilience.

(4) Using network analysis, proposing a more realistic behavior between the variables.

(5) Focus on intensive care professionals, with the potential to foster strategies.

## Introduction

According to the World Health Organization, on March 11^th^, 2020 the outbreak of the disease caused by the new coronavirus was characterized as a pandemic^1^. Since then, the impact of the disease on the population has been reflecting on the health services and on the work routines of the teams^2^.

In Brazil, the high incidence rates of the disease and the high mortality^3^ brought about an increased demand for critical care beds, impacting on the intensive care services. The increase in the workload in intensive care units and the greater need for Nursing professionals due to COVID-19 have already been evidenced in the literature^4^ ^-^ ^5^. In addition to the complexity and the requirement of human resources, acting directly in the care of patients can generate an increase in the emotional burden, arising from work and from the pandemic situation^6^.

In this context, Nursing professionals who work in intensive care are exposed, in addition to the risks inherent to the disease, to high workloads, unfavorable working conditions, experienced fear, difficulties providing care to patients and families, frequent contact with death and, also, psychological distress^2^.

Among the possible psychological impairments, the Burnout syndrome, mainly motivated by stressors in the workplace, consists of three dimensions: emotional exhaustion, depersonalization and low professional achievement. This can be characterized by difficulties in psychological, psychophysiological and behavioral adaptation which, mainly, affect professionals who perform their functions directly with people exposed to stressful situations^7^ ^-^ ^8^.

To cope with adverse situations and experiences at work, the professionals often need to seek individual tools that prevent the high burden of stress and harms to health. Among these, work resilience involves aspects such as creativity and innovation, hope, authenticity, high self-esteem for problem solving, critical thinking, autonomy, ability to interact with the environment, being proactive, dealing with unpredictability, managing stress and the support from the family and friends^9^. 

It is noteworthy that there are several concepts of resilience in the literature, but without a universal definition. Its construct involves physics and engineering, psychology, social aspects and health, as well as organizational relationships^9^. Despite its broad concept, it is known that most definitions of resilience include at least two points in common: the involvement of some form of adversity or challenge and then some degree of positive adaptation^10^. Therefore, they are characteristics that can facilitate the worker more positive responses to adversity and minimize the risk of illness^9^.

Inverse relationships between the Burnout syndrome and resilience have already been reported in the literature^11^ ^-^ ^12^ at the time prior to the pandemic, in which nurses with high resilience scores showed lower prevalence of the Burnout syndrome, acting as a partial mediator to this outcome^12^. However, the factors associated with nurses’ resilience are still questioned^13^.

A number of studies^11^ ^-^ ^15^ evidence resilience as an important protective factor in the face of the adversities. However, there is a gap in knowledge linked to the fact that investigations on this topic are mostly based on scales not specific to the work and health environment^11^ ^-^ ^15^, presenting uncertainties in the understanding of the factors that involve resilience in the work of these professionals.

In view of the above, the following is asked: Which is the relationship between the Burnout dimensions and the work resilience of intensive care Nursing professionals in the COVID-19 pandemic? Therefore, this study aims at analyzing the relationship between the Burnout dimensions and the work resilience of intensive care Nursing professionals in the COVID-19 pandemic at four hospitals from southern Brazil.

## Method

### Study design and location

This is a cross-sectional and multicenter study, guided by the STROBE (Strengthening the Reporting of Observational Studies in Epidemiology) guideline, used to report observational studies^16^.

The study included four hospitals in the state of Rio Grande do Sul, Brazil, which were described as H1, H2, H3 and H4. Hospital H1 is a public and university institution, mostly providing care via the Unified Health System (*Sistema Único de Saúde*, SUS); hospital H2 is a teaching hospital, a reference in trauma and had a specific wing for COVID-19; hospitals H3 and H4 are general and university hospitals. All hospitals were references in the care of patients with COVID-19, in need for intensive care.

### Period

The data collection period was between August 3^rd^ and October 22^nd^, 2020.

### Population

The population of intensive care Nursing professionals was 1,127, with 900 technicians and 227 nurses. It is noteworthy that the nursing assistants are not part of the intensive care teams.

### Selection criteria

Workers from the Nursing teams of the intensive care units who were working in assistance in the COVID-19 pandemic during the data collection period were included. Those removed from function during the pandemic of COVID-19 were excluded. 

### Sample definition

All professionals were invited to the study. Non-probabilistic sampling was used. Sample calculation took into account the network analysis, which estimates the partial correlation between the variables and the number of variables included in the analysis. The sample size exceeds the number of variables in the model (*n*= 18) and has the same number of non-redundant elements in the partial correlation matrix [(18*17/2)]^17^, accounting for a total of 153 participants.

### Study variables

The questionnaire contained sociodemographic information (gender, age, number of children, marital status, skin color, schooling level), health (increased alcohol consumption during the pandemic, smoking, physical activities, sleep quality, quality of the eating habits), work (institution, time in the institution, time in the sector, time in the profession, position, employment contract, other employment contract, work shift, relocation in the pandemic, care for patients with COVID-19, perception of exposure to the risk for COVID-19, work leave in the pandemic, days away from work, perception of the impact of the pandemic on physical health and mental health), minor psychological disorders (MPD)^18^, Burnout syndrome^7^ and resilience at work^9^.

The employment contract variable included employees, who respond to the rules established by the Consolidation of Labor Laws (*Consolidação das Leis do Trabalho*, CLT), with formal registration; temporary, with a contract of defined duration and statutory, referring to public servants.

The perception of sleep and eating habits quality were evaluated using a five-point Likert scale, varying from 1 (very poor) to 5 (excellent). To assess the perception of the impact of the pandemic on physical health and mental health, a five-point Likert scale was used, varying from 1 (no impact) to 5 (intense impact).

### Instruments used to collect the information

To evaluate the Burnout syndrome, the Maslach Burnout Inventory (MBI), validated in Brazil, was used^7^. The instrument has a five-point Likert scale and 22 questions. Nine questions assess emotional exhaustion (questions 1, 2, 3, 6, 8, 13, 14, 16 and 20), five assess depersonalization (questions 5, 10, 11, 15 and 22) and eight assess professional achievement, with an inverse score (questions 4, 7, 9, 12, 17, 18, 19 and 21)^7^. The answers indicated are added up and divided, in order to obtain the arithmetic mean of the scores in each dimension. The variables of the inventory’s three subscales are presented independently and can be measured and analyzed separately^7^ ^,^ ^19^.

To assess work resilience, the Resilience at Work - RAW Scale Brazil 20 was used, developed in Australia^10^ and validated in Brazil^9^. The scale has seven domains: Living authentically (questions 1, 2 and 3), Finding vocation (questions 5, 6, 7 and 8), Keeping the balance (questions 9, 10 and 11), Managing stress (questions 13, 14, 15 and 16), Interacting cooperatively (questions 17 and 18), Staying healthy (questions 20 and 21), Building networks (questions 23 and 24). The answer options vary from strongly disagree (0) to strongly agree (6). Questions nine and 11 have inversely scored items. It is noteworthy that the instrument includes 25 questions, with the option of using the versions with 20 or 25 items of the scale, both validated in Brazil. The general Cronbach’s alpha of the RAW Scale Brazil 20 remained at 0.79 and, for the scale’s domain, it varied from 0.49 to 0.85. Confirmatory factor analysis showed factor loadings ≥0.30^9^. The general score of the scale was used to verify the correlation between the variables. Due to copyrights, the analysis modality requires a request through the http://www.workingwithresilience.com.au website.

To track minor psychological disorders, the Self-Reporting Questionnaire (SRQ-20) was used, which contains 20 dichotomous questions about depressive, anxious and psychosomatic symptoms that occurred in the last 30 days from the answer. The score of one (1) indicates presence of symptoms and zero (0), absence. Seven or more affirmative answers are considered to identify minor psychological disorders^18^ ^,^ ^20^.

### Data collection

The workers were invited through the institutional email service, receiving the research instrument, via Google Forms and the Free and Informed Consent Form (FICF). The form received provided access to the FICF first. Subsequently, it was subdivided into sections: general worker’s data (sociodemographic and health issues), information about work (labor issues), Maslach Burnout Inventory, Self-Reporting Questionnaire and the Resilience at Work Scale - 20, constituting 93 mandatory questions. Prior evaluation of the questionnaire was performed to identify possible failures and estimate the response time.

As a strategy during data collection, visits were made to the units to invite the professionals who had not been granted access to the research instrument and also to remind them about the study.

In view of the limitations related to the critical pandemic period, it was not possible to carry out supervision tasks during completion of the forms. However, all professionals were instructed to answer them.

### Data treatment and analysis

The data were stored in Excel^®^ spreadsheets and later transferred to the SPSS^®^ Statistical Package for the Social Sciences, SPSS Inc, Chicago), version 18.0 for Windows, and to the JASP platform - Jeffreys’s Amazing Statistics Program (version 0.14.1)^17^. A descriptive and inferential statistical analysis was performed.

Distribution of the variables was verified using the Shapiro-Wilk test, considering p≤0.05 to indicate non-adherence to normal distribution. The parametric variables (age and time working in the profession) were described as mean and standard deviation. The other continuous, non-parametric variables were described in medians and interquartile intervals. The data obtained by means of the Likert scale were described in medians and interquartile ranges. The categorical variables were presented through of absolute and relative frequencies.

To verify the correlation between the variables, bivariate Spearman correlations were performed, suitable for analyses that include ordinal and continuous variables. To evaluate the difference between two groups and the Burnout syndrome domains (Emotional Strain, Depersonalization and Professional Achievement), the Mann-Whitney test was used and, for more than two groups, Kruskal-Wallis and Dunn. Therefore, it was verified which independent variables (sociodemographic, health and work-related variables) presented a significant relationship (p<0.05) with at least one of the dependent variables.

To investigate the relationship between Burnout and resilience at work, a network analysis was carried out, which comprises two stages: the first consists in estimating the matrix of regularized partial correlations (that is, with values close to zero, set at zero) through of the Graphical Least Absolute Shrinkage and Selection Operator (GLASSO) algorithm and the second stage, in which the partial correlation matrix is represented in a two-dimensional plane in a graphic object(17). The partial correlations are estimated using the inverse matrix (m-1) of the bivariate correlation matrix, which, in the JASP software, are calculated using the “cor_auto” function that considers the measurement and distribution level of each pair of variables (e.g. Pearson, Spearman, dot-biserial, polyserial, etc.).

The resulting non-directional network is formed by vertices, representing the variables investigated, and by edges, representing the partial correlations among them. The partial correlations can vary in magnitude (more or less thick lines) and in positive (blue) or negative (red) direction^21^. This analysis identifies the associations between two variables after controlling the effect that all others have on them; thus increasing the certainty of the inferences presented in the previous analyses.

### Ethical aspects

The study was approved by the Research Ethics Committee for each institution under study, as well as by the National Research Ethics Commission (Comissão Nacional de Ética em Pesquisa, CONEP), under opinion number 4,152,027 and CAAE: 33105820.2.0000.0008. The ethical precepts were respected according to Resolution 466/12 of the National Health Council (*Conselho Nacional de Saúde*, CNS)^22^. Clarifications about the study were transcribed in the Free and Informed Consent Forms and made available to the participants. Completion of the instrument was considered as acceptance to participate in the research.

## Results

The highest percentage of the participants corresponded to the female gender (78.4%; n=120). The mean age was 38.41 ± 7.42 years old and the median for children was 1 (0-2). Regarding marital status, 75.2% (n=115) were married or had a partner. The workers stated being white- (79.6%; n= 121), brown- (11.2%; n=17) and black-skinned (9.2%; n=14). The level of vocational/technical training represented 43.8% (n=67), followed by specialization/residency (30.7%; n=47), master’s/PhD degree (16.3%; n=25) and undergraduate degree (9.2%; n=14). 

Regarding life habits, 30.1% (n=46) reported an increase in alcohol consumption during the pandemic and 92.2% (n=141) were non-smokers. Physical activity was reported by 26.8% (n=41) of the workers. In addition to that, the perception of sleep quality had a median of 3 (2-4).

The work-related characteristics of intensive care Nursing professionals are presented in Table 1. Furthermore the perception of the impact of the pandemic on health had a median of 4 (3-4) for physical health and 4 (3-5) for mental health. Regarding work leaves during the pandemic, 43.1% of the cases (n=66) were due to suspected COVID-19 and 13.1% (n=20) due to confirmation of the disease.

**Table 1:** Work-related characteristics of intensive care Nursing professionals (N=153). Rio Grande do Sul, Brazil, 2020

Variables	n=153
Institution^*^	
Hospital 1	87 (56.9)
Hospital 2	27 (17.6)
Hospital 3	25 (16.3)
Hospital 4	14 (9.2)
Working time in the institution in months^†^	33 (5-123.5)
Time in the profession in months^‡^	145.55 ± 85.6
Position^*^	
Nurse	69 (45.1)
Nursing technician	84 (54.9)
Employment contract^*^	
CLT^§^	108 (70.6)
Temporary	42 (27.5)
Statutory	3 (1.9)
Working time in the sector in months^†^	33 (4-105)
Other employment contract^*^	
No	121 (79.1)
Yes	32 (20.9)
Work shift^*^	
Day	89 (58.2)
Night	64 (41.8)
Relocated to another sector/unit during the COVID-19 pandemic^*^	
No	104 (68)
Yes	49 (32)
Assistance to patients with COVID-19^*^	
No	5 (3.3)
Yes	148 (96.7)
Exposure to risk of the disease^†^	4 (3-5)
Work leaves during the pandemic^*^	
No	100 (62.7)
Yes	53 (37.3)
Days of work leaves^†^	7 (4-14)

^*^n*(f)*; ^†^Median and 25^th^ and 75^th^ percentiles; ^‡^Mean ± Standard Deviation; ^§^CLT = Professionals in a labor regime that responds to the rules established by the Consolidation of Labor Laws (CLT)

Regarding the workers’ psychological health, the prevalence of minor psychological disorders was 54.9% (n=84). In relation to Burnout, 11.1% (n=17) of the workers had the syndrome. Regarding the Burnout domains, 28.8% (n=44) presented emotional exhaustion; 39.9% (n=61), depersonalization and 26.1% (n=40), low professional achievement. Emotional exhaustion was low in 21.6% (n=33), moderate in 49.7% (n=76) and high in 28.8% (n=44). Depersonalization was low in 18.3% (n=28), moderate in 41.8% (n=64) and high in 39.9% (n=61). Regarding professional achievement, it was low in 24.2% (n=37), moderate in 49.7% (n=76) and high in 26.1% (n=40).

**Figure 1:**
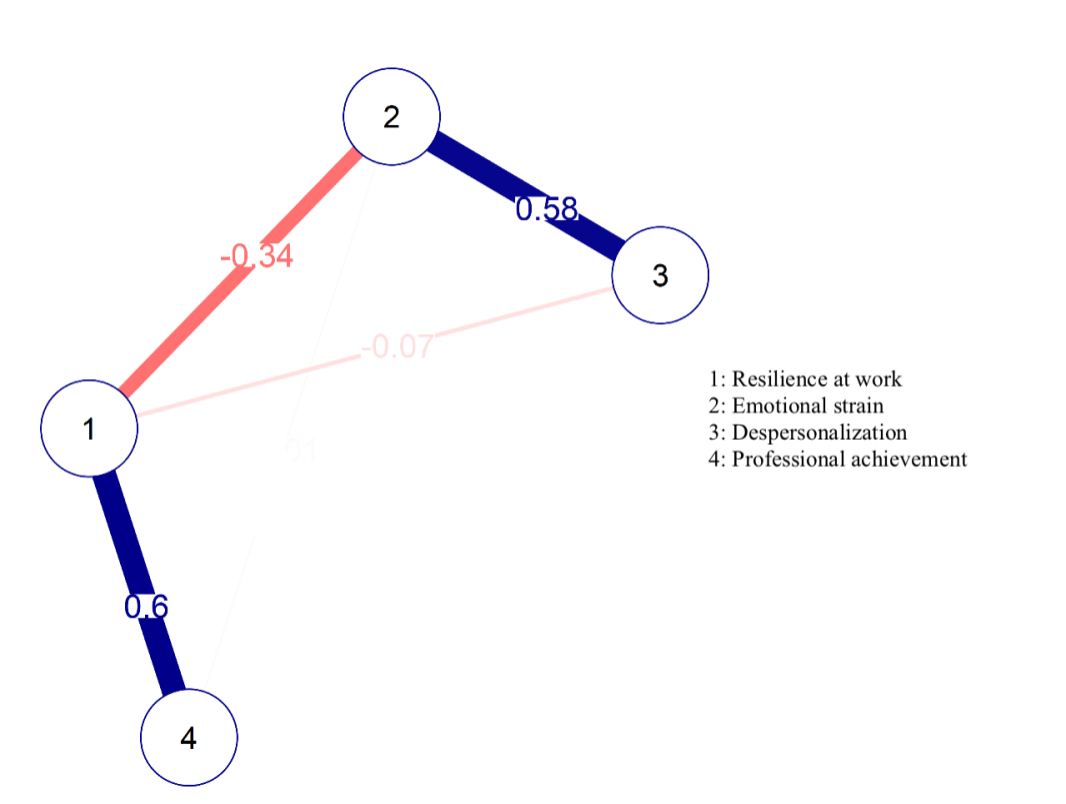
Network of correlations between the Burnout dimensions and resilience at work. Rio Grande do Sul, Brazil, 2020

The presence of minor psychological disorders was correlated with emotional exhaustion (r=0.632; p=0.01), depersonalization (r=0.477; p=0.01) and low professional achievement (r=-0.450; p=0.01). Resilience at work was inversely correlated with emotional exhaustion (r=-0.545; p=0.01) and depersonalization (r=-0.419; p=0.01) and directly correlated with professional achievement (r=0.680; p=0.01). There was a negative correlation between resilience at work and minor psychological disorders (r=-0.675; p=0.01). The network of correlations between the Burnout dimensions and resilience at work is represented in Figure 1.

The vertices represent variables and the edges represent positive (blue color) and negative (red color) associations

It is noted that depersonalization is driven by emotional exhaustion. Professional achievement directly interferes with resilience at work. Resilience at work proved to be a protective factor against emotional exhaustion.

The socio-occupational variables, with a statistically significant difference with the Burnout dimensions and/or with resilience at work, were included in the network analysis.

Emotional exhaustion was, significantly, related to time working in the institution (p=0.005), working time in the sector (p=0.009), perception of the impact of the pandemic on physical (p<0.001) and mental (p<0.001) health, days of work leaves (p=0.024), position (p=0.007), employment contract (p=0.01), perceived risk of exposure to COVID-19 (p<0.001), work shift (p=0.001) and sleep quality (p<0.001).

Depersonalization was, significantly, related to the practice of regular physical activity (p=0.01), working time in the institution (p=0.043), the perception of the impact of the pandemic on physical (p=0.001) and mental (p=0.001) health, perceived risk of exposure to COVID-19 (p<0.001), sleep quality (p=0.02) and work shift (p=0.01).

Professional achievement was, significantly, related to time working in the institution (p=0.005), the perception of the impact of the pandemic on mental health (p<0.001), days of work leaves (p=0.024), employment contract (p=0.009), need to distance from work (p=0.035) and work shift (p=0.001).

Resilience at work was, significantly, related to the perception of the impact of the pandemic on mental health (p<0.001), employment contract (0.024), sleep quality (0.012) and work shift (p=0.001).

**Figure 2:**
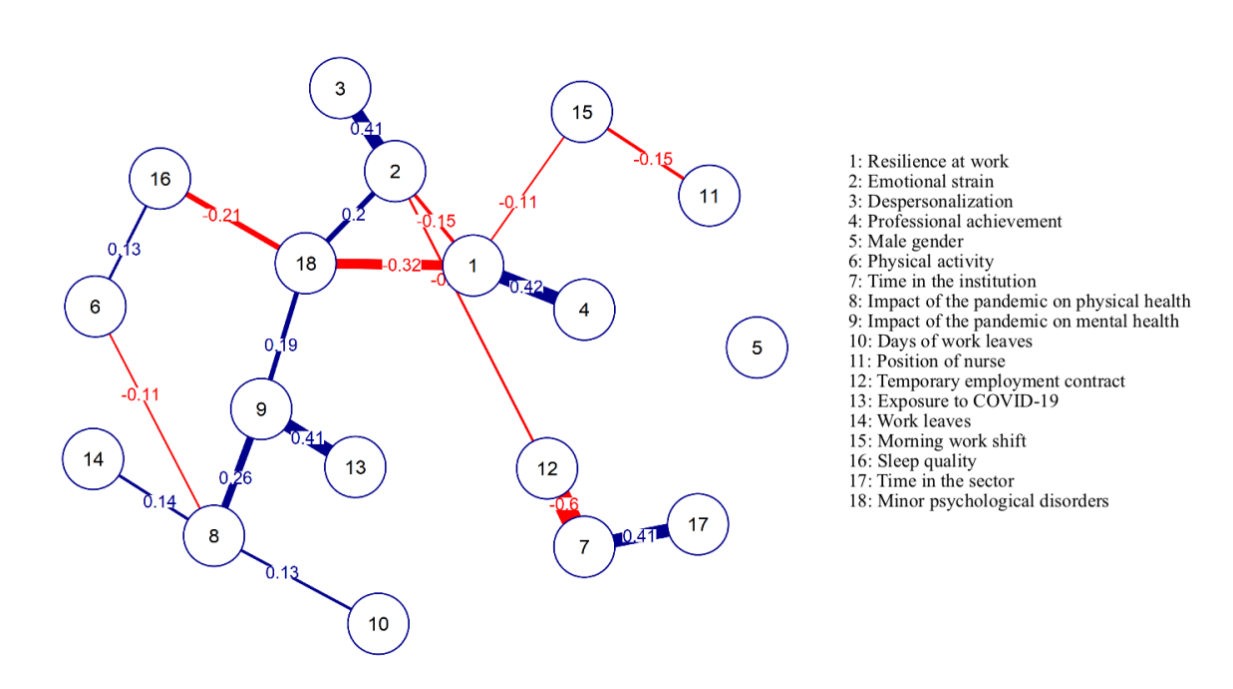
Network of partial correlations between the burnout dimensions, resilience at work and socio-occupational variables. Rio Grande do Sul, Brazil, 2020

Analyzing the network of partial correlations (Figure 2), some relevant groups in this system stand out. Minor psychological disorders interfered with the impact of the pandemic on mental health, aggravated by emotional exhaustion. Depersonalization is driven by emotional exhaustion, with a direct and high magnitude relationship. The perception of the impact of the pandemic on mental health showed, with a high association strength, to be influenced by the perception of exposure to COVID-19 and the perception of the impact of the pandemic on physical health. The perceptions about physical health showed to be driven through variables about the workers’ need and time of work leaves. With a weaker magnitude and an inverse relationship, the professionals who did not perform physical activities and reported having poor sleep quality had a more negative perception of the impact of the pandemic on physical health, in addition to a greater relationship with minor psychological disorders. Emotional exhaustion also presented an inverse and weak relationship with work-related variables such as having a temporary job, shorter time in the institution and in the work sector, that is, with lower rates for this domain.

Resilience proved to be a protective factor against minor psychological disorders and emotional distress. Resilience also presented a strong positive correlation with professional achievement. The morning shift professionals presented an inverse correlation to resilience at work and to the position of nurse. The gender variable did not interfere with the network model. 

The vertices represent variables and the edges represent positive (blue color) and negative (red color) associations

Centrality measures allow us to identify the most relevant variables on the network of regularized partial correlations, with the greatest potential to provide changes. Therefore, according to the centrality table (Table 2), the variable that presented the greatest influence on the network of correlations was the perception of the impact of the pandemic on mental health.

**Table 2:** Centrality measures of the network of regularized partial correlations (N=153). Rio Grande do Sul, Brazil, 2020

Variables	Expected influence
Resilience at work	-0.805
Emotional distress	1.005
Depersonalization	0.756
Professional achievement	0.580
Male gender	-0.674
Physical activity	-0.515
Time in the institution	-0.688
Impact of the pandemic on the physical health	0.949
Impact of the pandemic on the mental health	2.166
Days of work leaves	0.005
Position of nurse	-0.809
Temporary employment contract	-1.855
Exposure to COVID-19	1.020
Work leaves	0.448
Morning work shift	-0.973
Sleep quality	-0.468
Time in the sector	0.683
Minor psychological disorders	-0.824

## Discussion

The findings of this study evidenced resilience at work as a protective factor in the presence of high levels of emotional exhaustion and depersonalization, domains with the highest scores in the Burnout syndrome.

A number of studies^23^ ^-^ ^24^ carried out with intensive care professionals in European countries indicate a higher prevalence of Burnout. However, at the international level, identification of the syndrome is performed in a different way, without considering the three domains for the characterization of Burnout. In a Brazilian study carried out before the pandemic, high levels of emotional exhaustion and depersonalization were also found, justified by the high workload of professionals^25^.

The work environment of intensive care units is characterized by high technological density and complexity of care, requiring from the professional technical and specific knowledge, quick thinking, constant scientific updating and emotional balance to face adversities. During the COVID-19 pandemic, an Italian study^26^ evidenced that these professionals’ workload increased significantly, linked to complex procedures, such as pronating patients and the use of Extracorporeal Membrane Oxygenation (ECMO), to the pathophysiology of the disease^27^, to prolonged mechanical ventilation, to increased use of vasoactive drugs and to the occurrence of adverse events^28^. With the advent of the pandemic, factors such as the professionals’ gowning and degowning, the feasibility of communication between family members and patients, and the management of *delirium* with a higher incidence should also be considered when measuring the workload^29^.

All these factors, inherent to work in intensive care, reveal the profound changes and adaptations that have taken place in the services in the face of the pandemic, becoming stressors and potentially aggravating health. The perception of the impact of the pandemic on physical and mental health and the perception of exposure to the disease showed to interfere with mental health, in the presence of minor psychological disorders and high emotional exhaustion. A review study pointed out that the most frequent feeling among health workers during the pandemic was fear and that negative perceptions related to mental health were linked to insomnia, psychological distress, Burnout, anxiety and depressive symptoms. As for physical health, the manifestations were limited to symptoms resulting from COVID-19 infection^30^. As a result, negative feelings and psychological health problems do not seem to be considered an issue linked to physical health.

A qualitative study^31^, about the nurses’ emotions and perceptions in the face of the pandemic, reveals feelings of concern, tension and fear in the face of exposure to infection, also related to the possibility of COVID-19 contamination of their family members. Anguish, despair, sadness, frustration and anxiety were also observed through the participants’ testimonies, corroborating our findings that point to the relationship between mental health issues and the perception of the impact of the pandemic on health. The authors highlight the importance of self-knowledge and the detection of warning signs and manifestations, to develop actions aimed at coping with and reducing such feelings^31^.

The perception of the impact of the pandemic on physical health was related to the need to distance from work and to longer periods of time away from work. In addition to leaves due to COVID-19, mental health issues such as somatization can be related to high stress levels, which are often only considered when they interfere with physical health to justify the need for a work leave. A study with the objective of performing a psychological intervention in health professionals evidenced that there is resistance on the part of many workers to accept psychological support. The authors point out that, despite showing excitability, irritability, unwillingness to rest and signs of psychological distress, the nurses refused any psychological help and stated that they had no problems. Among the staff, this refusal of psychological support was linked to a desire for uninterrupted rest, sufficient protective supplies, and the need for training in psychological skills to deal with the patients’ anxiety, panic and other emotional problems, as well as the support provided by the mental health team to these patients^32^.

Therefore, when the impact of work on health is not managed, it can lead to physical and psychological harms. In addition, the relationship between the presence of health problems and greater susceptibility to emotional exhaustion was also evidenced in a study carried out with Portuguese nurses during the pandemic^33^, corroborating the relationship between physical and psychological health.

It is noteworthy that our data come from a critical period of the pandemic, at the peak of the number of cases, with overcrowding of intensive care units and in the absence of vaccines, which reflects in high exposure of the professionals. According to the Federal Nursing Council^34^, 44,441 nurses, technicians and assistants were distanced from work in 2020, after infection with the new coronavirus. In addition to the factors that involve workers’ exposure, exhaustion also became a concern due to the increased risk of errors with personal and assistance care measures, considering the extreme fatigue of the teams due to the long period of work and stress^34^.

Sleep quality and performance of physical activities showed a positive impact on physical health and a protective factor for minor psychological disorders. It is known that sleep has a function of physical restoration, conservation, energy and protection^35^ and that the practice of physical exercise is fundamental for maintaining health. Due to the pandemic and the high number of leaves, many professionals faced double working hours, as well as high workload and stress, factors that can affect sleep quality. In addition to that, the need for a lockdown and social isolation restricted leisure activities and sports, harming health and quality of life^36^.

A study carried out in China indicated that working on the front lines in the fight against the pandemic was, significantly, associated with increased anxiety and stress levels, with a negative influence on the professionals’ sleep quality^37^. Greater susceptibility to depressive symptoms, anxiety, stress and sleep-related problems in women and nurses has also been reported, considering the places with the greatest focus of the pandemic as a heightened risk for these symptoms^38^. The authors reinforce the link between sleep and mental health, in which insomnia can be related to psychopathologies such as depression or post-traumatic stress disorder, after a stressful event. In addition to that, people who suffer from insomnia have greater difficulties dealing with daily stressors^39^.

Regarding the practice of physical activities, a study showed a lower risk of illness for the group that exercised regularly^40^. Performance of physical exercises is capable of producing beneficial effects for physical and mental health, being considered effective in the prevention of mood disorders and neurodegenerative diseases through mechanisms involved in the organs-brain signaling axis^41^.

Resilience at work proved to be a protective factor for the mental health variables, such as presence of minor psychological disorders, emotional exhaustion and depersonalization. A study carried out in China, with front-line nurses at the peak of the COVID-19 pandemic, revealed the association between resilience and Burnout in this population. Resilience showed significant negative correlations with burnout, emotional exhaustion, depersonalization and reduced personal achievement. Negative affects such as fear, nervousness, irritability, hostility and shame and positive affects such as enthusiasm, attention, pride, hope and contentment, were mediators in the Burnout domains, whose study proposes to establish resilience strategies for front-line health professionals to reduce burnout^42^.

A study conducted with front-line female nurses from Philippines also evidenced that increased levels of personal resilience, organizational support and social support in nurses were associated with reduced anxiety levels related to COVID-19^43^.

Our findings evidenced a direct relationship between emotional exhaustion and longer time in the profession. In the literature, professional experience tends to improve sound awareness in problem solving, which can increase confidence in the professional actions, inducing less stress and anxiety^33^. However, a number of Italian authors, who reported their perspectives within an Intensive Care Unit during the pandemic, highlight that older and more experienced nurses needed to train new Nursing professionals, due to care complexity. Therefore, the responsibility was assigned to guarantee a high level of care and, at the same time, support less experienced Nursing colleagues, making their work routines more intense and stressful^44^.

As for work shift, the morning professionals presented an inverse relationship to resilience at work. According to the literature, professionals with low levels of resilience present a higher risk of illness and this is, mainly, related to night shift professionals. The factors related to the risk of illness mainly include sleep disorders, long working hours and agreement with the chronotype, in addition to the practice of physical activity as a protective factor^40^ ^,^ ^45^ ^-^ ^46^. It is noteworthy that the professionals working the morning shift can also be affected by these factors, as they assume the first demands of the patients in the day, have greater contact with the family members and participate in activities established for the morning shift. In addition to that, the morning shift teams have contact with a greater number of professionals, which can be related to problem solving and conflict management.

In relation to the professional position, a review study on resilience in health workers highlighted that Nursing professionals have lower resilience scores when compared to other professions, and that front-line professionals also have lower resilience rates. The authors report that the nurses with higher resilience scores had fewer negative mental health results, including anxiety, depression and post-traumatic stress disorder^47^. An important factor to increase the professionals’ resilience and the impulse to overcome the challenges facing the pandemic was the presence of teamwork^48^. Therefore, providing ways to build resilience among health professionals with a focus on increasing resilience in nurses is encouraged, seeking to understand the impact of interventions on the resilience scores and on coping with emotional distress^49^.

Furthermore, managers should prioritize building personal resilience among front-line nurses, as greater personal resilience was associated with less anxiety motivated by COVID-19. Positive coping strategies and nurses’ self-efficacy should be encouraged, in addition to providing adequate organizational support, which involves maintaining a safe work environment, availability of quality Personal Protective Equipment in sufficient numbers, supplies to prevent infections, provision of accurate and timely information about the disease and implementation of training sessions relevant to COVID-19^43^.

As limitations, the cross-sectional design does not allow monitoring the individuals to verify the impacts of the pandemic on mental health, requiring a cohort study. There are also few investigations that assess resilience at work with the instrument used, creating difficulties in corroborating some results. In our study, only healthy workers were included, as well as an important percentage of new workers in the profession and in the institution, with attenuated risk factors for developing Burnout syndrome in the first moment of the pandemic, when data collection took place.

This study has important implications about strategies that can be made available to Nursing professionals in coping with COVID-19 and any other possible pandemic. The network analysis allows for a more realistic assessment of the variables that affect workers’ health and that may be associated with resilience at work. In addition to that, the advancement of knowledge about resilience at work is highlighted, as the scale applied to the professionals is still recent and not widespread, with the potential to fill gaps in knowledge.

## Conclusion

Resilience positively interferes in the emotional exhaustion and low professional achievement domains of Burnout. Emotional exhaustion is conducted through minor psychological disorders, with an impact on the workers’ physical and mental health variables. The level of exposure to COVID-19 influences the perception of the impact of the pandemic on mental health. Strategies that work on the professionals’ resilience can guarantee more positive responses to adversities and should be considered at the institutional level to minimize the occurrence of Burnout among the intensive care Nursing team.
